# Pan-American Medical Congress

**Published:** 1892-08

**Authors:** 


### PAN-AMERICAN MEDICAL CONGRESS.

(*Official Bulletin.*)

### BY-LAWS OF THE BOARD OF TRUSTEES.

THE committee appointed to prepare by-laws for the government of the Board of Trustees, submitted the following report :

#### I. — OFFICERS.

SEC. 1. The officers shall consist of a Chairman, a Secretary, who shall act as assistant secretary to the Secretary-General of the Congress, and a Treasurer.

§ 2. The Chairman shall be elected by ballot ; the Secretary shall be appointed by the Chairman, on recommendation of the Secretary-General ; the Treasurer shall be the person elected Treasurer of the Congress. These officers shall perform the duties usually performed by such officers.

§ 3. These officers shall hold office during the continuance of the corporation.

#### II. — TREASURER.

The Treasurer shall give a good and sufficient bond, in the sum of $25,000. He shall receive all registration fees, and all other moneys paid to the credit of the Congress, and shall disburse the same on vouchers approved by the Board of Trustees, signed by the Secretary-General and the Chairman of the Executive Committee.

#### III. — EXECUTIVE COMMITTEE.

There shall be an Executive Committee, consisting of seven Trustees, who shall conduct the business of the Trustees in the intervals between the meetings of the Trustees, and shall report in full at each meeting.

### IV.—The Committee of Arrangements.

The Committee of Arrangements shall consist of such number of physicians of the City of Washington, D. C., as may be determined upon and appointed by the President of the Congress, one member of which committee shall be designated by him as chairman. The Committee of Arrangements shall have charge of the facilities for registration, shall provide for transportation rates for physicians and their families attending the Congress, shall arrange for adequate hotel accommodations, shall arrange for an entertainment of the Congress, and issue the official programme of the meeting. All expense thus incurred shall be paid out of the funds previously appropriated for the purpose by the Executive Committee, payment of the same to be made by the Treasurer on the official voucher of the Congress countersigned by the Chairman of the Committee.

### V.—Committee on Ways and Means.

A committee, not to exceed one hundred in number, shall be appointed by the President of the Congress, and shall be known as the Committee on Ways and Means, whose duty it shall be to devise and execute methods by which the necessary expenses of the Congress may be defrayed.

### VI.—Expenditures.

Sec. 1. The Executive Committee may expend a sum not to exceed $3,000 prior to the meeting of the Congress, which sum shall include the expense of a stenographer for the Secretary-General. All bills contracted shall be paid as soon as funds in the hands of the Treasurer will permit.

§ 2. The Executive Committee shall designate all expenditures by the various officers before the same shall be contracted, providing that expenses already incurred by the Committee on Permanent Organization shall be paid out of any funds in the hands of the Treasurer of the Congress.

### VII.—Meetings.

The first meeting of the Trustees shall be held in Detroit, Mich., on June 8, 1892. Thereafter all meetings shall be held at such time and place as the Executive Committee shall designate.

### VIII.—Vouchers.

The form of vouchers hereunto annexed shall be the form used in the payment of all accounts by the Treasurer, and shall be in duplicate, one for the Treasurer and one for the Secretary-General.

HENRY D. HOLTON,
W. T. BRIGGS,
JOHN B. ROBERTS.
*Committee on By-laws.*

FORM OF VOUCHER.

*ORIGINAL.—For the Treasurer.*
*DUPLICATE.—For the Secretary-General.* *No...........*

THE PAN-AMERICAN MEDICAL CONGRESS.

*To......................Dr.*

*Date.*

*ORIGINAL.*

*Total in words, $......*

I CERTIFY the above account is correct and true ; that the same is according to contract on file, or pursuant to order of........under date of....189 , and duly authorized by the Executive Committee appointed by the Board of Trustees of the Pan-American Medical Congress.

.........................*Secretary-General.*

The above account is hereby approved and the Treasurer instructed to pay the same.

Dated at......, ....189 . ........................
*Chairman of the Executive Committee.*

*Received of* ABRAHAM M. OWEN, Treasurer, his cheque for........ dollars and........cents, in full of the above account.

.........................

[ENDORSEMENT.]

THE PAN-AMERICAN MEDICAL CONGRESS.

*VOUCHER.*
No....

ORIGINAL.

Name.............. For ............ $........
Duplicate signed and returned to the Secretary-General. .
.....................A. D. 189 .
.................... *Treasurer.*

---

*ORIGINAL—For the Treasurer.*
*DUPLICATE—For the Secretary-General.* *No........*

THE PAN-AMERICAN MEDICAL CONGRESS.

*To......................Dr.*

*Date.*

*DUPLICATE.*

*Total in words, $......*

I CERTIFY the above account is correct and true ; that the same is according to contract on file, or pursuant to order of........under date of....189 , and duly authorized by the Executive Committee appointed by the Board of Trustees of the Pan-American Medical Congress.

............................*Secretary-General.*

The above account is hereby approved and the Treasurer instructed to pay the same.

Dated at......, ....189 . ........................

*Chairman of the Executive Committee.*

*Received of* Charles A. L. Reed, Secretary-General of the Pan-American Medical Congress, original certified and approved voucher for......dollars and......cents, in full of the above account.

........................

*Treasurer.*

[Endorsement.]

THE PAN-AMERICAN MEDICAL CONGRESS.

*VOUCHER.*

No....

DUPLICATE.

Name.............. For............ $........

Original duly certified and forwarded to the Chairman of the Executive Committee.

.....................

*Secretary-General.*

Detroit, Mich., June 7, 1892.

At a meeting of the Trustees of the Pan-American Medical Congress, held at the Russell House, Detroit, Mich., June 8, 1892, the foregoing by-laws were adopted by unanimous vote.

A. WALTER SUITER, M. D.,
*Asst. Secretary-General for the Board of Trustees.*

